# Altered larval activation response associated with multidrug resistance in the canine hookworm *Ancylostoma caninum*

**DOI:** 10.1017/S0031182023001385

**Published:** 2024-03

**Authors:** Elise L. McKean, Emilia Grill, Young-Jun Choi, Makedonka Mitreva, Damien M. O'Halloran, John M. Hawdon

**Affiliations:** 1 Department of Microbiology, Immunology, and Tropical Medicine, The George Washington University, Washington, DC, USA; 2 Department of Biological Sciences, The George Washington University, Washington, DC, USA; 3 Infectious Diseases Division, Department of Medicine, Washington University School of Medicine, St. Louis, MO, USA; 4 McDonnell Genome Institute, Washington University, St. Louis, MO, USA

**Keywords:** *Ancylostoma caninum*, benzimidazole, dog hookworm, macrocyclic lactone, multiple anthelmintic drug resistant, pyrantel

## Abstract

Parasitic gastrointestinal nematodes pose significant health risks to humans, livestock, and companion animals, and their control relies heavily on the use of anthelmintic drugs. Overuse of these drugs has led to the emergence of resistant nematode populations. Herein, a naturally occurring isolate (referred to as BCR) of the dog hookworm, *Ancylostoma caninum*, that is resistant to 3 major classes of anthelmintics is characterized. Various drug assays were used to determine the resistance of BCR to thiabendazole, ivermectin, moxidectin and pyrantel pamoate. When compared to a drug-susceptible isolate of *A. caninum*, BCR was shown to be significantly resistant to all 4 of the drugs tested. Multiple single nucleotide polymorphisms have been shown to impart benzimidazole resistance, including the F167Y mutation in the *β*-tubulin isotype 1 gene, which was confirmed to be present in BCR through molecular analysis. The frequency of the resistant allele in BCR was 76.3% following its first passage in the lab, which represented an increase from approximately 50% in the founding hookworm population. A second, recently described mutation in codon 134 (Q134H) was also detected at lower frequency in the BCR population. Additionally, BCR exhibits an altered larval activation phenotype compared to the susceptible isolate, suggesting differences in the signalling pathways involved in the activation process which may be associated with resistance. Further characterization of this isolate will provide insights into the mechanisms of resistance to macrocyclic lactones and tetrahydropyrimidine anthelmintics.

## Graphical abstract

**Figure:**
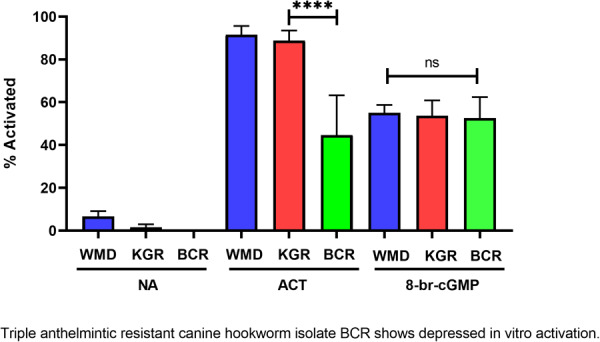

## Introduction

Currently, parasitic gastrointestinal nematodes (GINs) of medical and veterinary importance are primarily controlled using anthelmintic drugs that remove the adults from the host. The reliance on anthelmintics has resulted in the widespread development of resistant nematodes, especially in livestock parasites, some of which are resistant to 1 or more major classes of anthelmintic drugs (Kaplan, 2004, 2020; Wolstenholme *et al*., 2004; Kaplan and Vidyashankar, 2012). Recent studies by us (Kitchen *et al*., 2019) and others (Hess *et al*., 2019; Jimenez Castro *et al*., 2019) have reported the emergence of multi-anthelmintic drug-resistant (MADR) *Ancylostoma caninum* hookworms which have spread to the general companion dog population from retired greyhounds (Jimenez Castro *et al*., 2019; Jimenez Castro and Kaplan, 2020; Balk *et al*., 2023; Venkatesan *et al*., 2023). Most of these hookworm isolates are resistant to the 3 major classes of anthelmintics used for treatment: the benzimidazoles (BZ), the macrocyclic lactones (ML) including ivermectin (IVM), and the imidazothiazoles/tetrahydropyrimidines levamisole and pyrantel (PYR). Of these, a molecular mechanism of resistance is known only for BZ resistance. In GINs infecting livestock, resistance is associated with mutations in 1 of 3 amino acids in isotype 1 of the *β*-tubulin gene: a phenylalanine to tyrosine mutation in codons 167 or 200, or a glutamate to alanine or leucine change in codon 198, with the 167 and 200 mutations occurring more frequently (Kwa *et al*., 1994; Silvestre and Cabaret, 2002; Ghisi *et al*., 2007; Vercruysse *et al*., 2011). Recently, several additional mutations in codon 198 found at low frequencies in parasitic nematode populations have been shown to confer resistance to BZ in *Caenorhabditis elegans* (Avramenko *et al*., 2019; Dilks *et al*., 2020, 2021; Mohammedsalih *et al*., 2020). Resistance to benzimidazoles has been reported in populations of *A. caninum* across the country, with varying frequencies of the 167 alleles as well as the recently reported novel Q134H mutation (Jimenez Castro *et al*., 2019, 2021; Kitchen *et al*., 2019; Venkatesan *et al*., 2023). Both the F167Y and Q134H mutations were definitively confirmed to impart BZ resistance using *C. elegans* as a surrogate (Kitchen *et al*., 2019; Venkatesan *et al*., 2023). The mechanisms of resistance to IVM and PYR are currently unknown. Recently, several quantitative trait loci have been linked to ivermectin resistance in GINs, and the putative transcription factor *cky-1* has been identified as a possible mediator of IVM resistance (Choi *et al*., 2017; Doyle *et al*., 2019; Doyle *et al*., 2022, Laing *et al*., 2022). Pyrantel resistance in *A. caninum* has been associated with alterations of nematode acetylcholine receptor subunit expression levels (Kopp *et al*., 2009), but the underlying genetic mutation is unknown.

When third-stage infective larvae (iL3) of *A. caninum* are incubated with a host-mimicking signal *in vitro*, they resume feeding over a period of 24 h, which has been termed larval activation (Hawdon and Schad, 1990, 1991*a*; Hotez *et al*., 1993). Activation is thought to represent initial steps in the transition to the parasitic stage of the life cycle, and is analogous to feeding associated with recovery of the analogous *C. elegans* dauer larva from developmental arrest (Hawdon and Schad, 1991a; Hotez *et al*., 1993; Crook, 2014). The molecular components of the *A. caninum* activation pathway are highly conserved with those of the *C. elegans* dauer pathway, and include cGMP and insulin-like signalling (ILS) pathways (Tissenbaum *et al*., 2000; Hawdon and Datu, 2003; Brand and Hawdon, 2004; Gao *et al*., 2009; 2010; Kiss *et al*., 2009; Wang *et al*., 2009). Here a triple resistant isolate of *A. caninum* that exhibits an alternate activation phenotype compared to our susceptible isolate is described.

## Materials and methods

### Ethics statement

All experiments involving animals were conducted in strict accordance with the recommendations of the National Institute of Health (USA) Guide for the Care and Use of Laboratory Animals and the Animal Welfare Act (National Research Council, 2011; USDA, 2018). The animal protocol used in this study was approved by the Institutional Animal Care and Use Committee of The George Washington University (USA).

### Parasites and reagents

Wild-type laboratory isolate WMD and multidrug-resistant isolate KGR were described previously (Kitchen *et al*., 2019). The KGR isolate used here is resistant to thiabendazole (TBZ) and IVM. Multidrug-resistant BCR was isolated from a retired racing greyhound, which was raised in Kansas and raced in Florida, in October 2019. The dog had a history of recurring hookworm infections, and had been treated with Drontal Plus (praziquantel/pyrantel pamoate/febantel) prior to adoption. Infective third-stage larvae (iL3s) reared from eggs collected from feces of the infected dog were used to infect a hookworm naïve beagle. All isolates were maintained in beagles as described previously (Schad, 1982; Krepp *et al*., 2011). Thiabendazole, ivermectin, pyrantel pamoate, dimethyl sulfoxide (DMSO) and 8-bromo-cGMP were purchased from Sigma-Aldrich (St. Louis, MO, USA). Moxidectin (MOX) was a gift from Zoetis (Kalamazoo, MI, USA).

### Larval development assay

Larval development assays (LDA) were performed as described in Kitchen *et al*. (2019) with slight modifications. Briefly, stock concentrations of 10 mm IVM, 10 mm MOX and 10 mg mL^−1^ PYR were serially diluted 2-fold in 1% DMSO to concentrations twice the desired final concentration. For each drug concentration and control, 1.56 × 10^8^ cells of ampicillin-resistant OP50 and 100 L1s in nematode handling buffer BU (50 mm Na_2_HPO_4_, 22 mm KH_2_PO_4_, 70 mm NaCl, pH 6.8) (Hawdon and Schad, 1991b) with ampicillin in a volume of 250 *μ*L were added to duplicate wells of a 24-well tissue culture plate. In total, 250 *μ*L of the appropriate drug concentration or 1% DMSO for controls was added to the wells. The final concentrations of IVM were between 1000 and 1.95 nm, and the final concentrations of PYR were between 168.2 and 0.33 mm. Plates were incubated at 27°C for a minimum of 7 days, after which 100 *μ*L of Lugol's iodine was added to each well, and the number of larvae that had reached the iL3 stage and the number that did not reach the iL3 stage (L1 + L2) was counted. The percentage developing across all replicates was determined using the formula iL3_total_/((L1 + L2)_total_ + iL3_total_) × 100. The percentage of larvae developing to the iL3 was normalized to the average development of iL3 in control wells. Three biological replicates with 2 technical replicates for each concentration were performed for each hookworm isolate. The half maximal inhibitory concentration (IC_50_) was calculated by plotting the average percentage of larvae developing to the iL3 stage against the log of the drug concentration. The resulting curve was analysed using the sigmoidal dose–response (variable slope) function in GraphPad Prism (ver. 9, GraphPad Software, San Diego, CA, USA) to generate the IC_50_. Resistance ratios (RRs) at the IC_50_ concentrations for each drug were calculated by dividing the resistant isolate IC_50_ values by the corresponding wild-type values.

### Egg hatch assay

Egg hatch assays (EHAs) were performed as described (Jimenez Castro *et al*., 2019) except that 3-fold dilutions of TBZ between 50 and 0.002 mm were used, and Lugol's iodine was not added to wells before counting. A minimum of 4 biological replicates with 3 technical replicates per concentration were performed for each isolate.

### Fecal egg count reduction test (FECRT)

After 101 days of infection, the laboratory dog infected with the BCR isolate was treated orally with pyrantel pamoate (5 mg kg^−1^). Both prior to and following treatment, fecal egg counts (FEC) were regularly performed by homogenizing 2 g of feces in 60 mL of saturated salt solution and counting the eggs using a McMaster slide. The number of observed eggs was multiplied by 100 to determine the eggs per gram (EPG). The average EPG during the week prior to treatment and the average EPG on days 9, 10 and 11 post treatment were determined. A second dog infected with the KGR isolate was treated orally at day 37 post-infection with 5 mg kg^−1^ pyrantel pamoate, and the average EPG 3 days prior to treatment and on days 10, 11 and 12 post treatment were calculated. The equation ((pre-treatment FEC – post-treatment FEC)/(pre-treatment FEC)) × 100 to determine reduction in fecal egg count (FECR). Third-stage larvae from the first passage after pyrantel (BCR (P1)) were collected for genetic analysis.

### *β*-tubulin codon 167 relative allele frequency determination

The relative frequency of the *β*-tubulin codon 167 resistant and susceptible alleles was determined according to Kitchen *et al*. (2019). Briefly, genomic DNA was isolated from each isolate using the DNeasy Blood and Tissue Kit (Qiagen, Germantown, MD, USA). Separate quantitative polymerase chain reactions (PCRs) containing Brilliant II SYBR Green QPCR Master mix (Agilent Technology, Santa Clara, CA, USA), 10 ng of genomic DNA, a common reverse primer (5′-gctggcgccttcgccttttc-3′) and a forward primer designed to amplify either the susceptible wild-type allele (5′-gataggatcatgtcctcgtt-3′) or the resistant mutant allele (5′-gataggatcatgtcctcgta-3′) of the *tbb-iso-1* gene were performed on a CFX96 detection system (BioRad, Hercules, CA, USA) according to the manufacturer's suggested conditions. Relative allele frequencies were calculated using the formula:
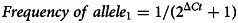
where ΔCt = (Ct of allele_1_-specific PCR)–(Ct of allele_2_-specific PCR). Ct values were the mean of at least 3 technical replicates (Germer *et al*., 2000).

As part of a larger study, we have sequenced the genomes of KGR, BCR, BCR (P1) and WMD isolates (data not shown). A pool-seq approach (Schlotterer *et al*., 2014; Choi *et al*., 2017) was employed to ascertain the allele frequencies of *β*-tubulin isotype 1 (*tbb-iso-1*) mutations F167Y (TTC>TAC) and Q134H (CAA>CAT) in KGR, BCR and WMD isolates. Only sites with a log-likelihood ratio of polymorphism greater than 22 (-p 22) were retained for allele frequency analysis. The sequences are available under BioProject PRJNA72585 (accession numbers SRR26411280 -SRR26411283).

### cDNA synthesis and *β*-tubulin isotype 1 (tbb-iso-1) sequencing

Total RNA was isolated from approximately 10 000 BCR iL3 using Trizol (ThermoFisher, Waltham, MA USA ) and cDNA synthesized as described previously (Kitchen *et al*., 2019). The *tbb-iso-1* cDNA was PCR amplified using primers SHH282 (5′-atgcgtgagatcgtgcatgt-3′) and SHH286 (5′- ctactcctcggggtaagcct-3′), and pooled reactions purified using the Nucleospin Gel and PCR Clean-up kit (Takara, San Jose, CA USA). Purified amplicons were Sanger sequenced by MCLAB (South San Francisco, CA ) using primers SHH282 (5′-atgcgtgagatcgtgcatgt-3′), SHH283 (5′-gcaaagaggcggaagg-3′), SHH284 (5′-caacagagaacgaagacatg-3′), SHH285 (5′-tttgctccactttcggc-3′) and SHH286 (5′- ctactcctcggggtaagcct-3′). The BCR *tbb-iso-1* cDNA sequence was submitted to GenBank with the accession number MZ889668.

### Larval activation assay

Infective L3 were recovered from coprocultures by a modified Baermann technique 7–10 days post-culture and stored for up to 3 weeks in BU at 22°C until used for activation studies. *Ancylostoma caninum* iL3 were activated under host-like conditions as described previously (Hawdon *et al*., 1999). Briefly, iL3 collected from coprocultures were decontaminated with 1% (v/v) HCl in BU buffer for 30 min at 22°C. Approximately 250 iL3 were incubated at 37°C, 5% CO_2_ for 24 h in 0.1 ml RPMI1640 tissue culture medium supplemented with 25 mm HEPES pH 7.0 and antibiotics (RPMI-c) in individual wells of 96-well microtiter plates (Hawdon and Schad, 1990). Activated L3 were stimulated by inclusion of 10% (v/v) canine serum and 15 mm S-methyl-glutathione (GSM; Sigma) or 10 mm 8-bromo-cGMP (Sigma) dissolved in RPMI-c (Hawdon *et al*., 1995; Hawdon and Datu, 2003). Negative control (non-activated) L3 were incubated in RPMI-c without the stimuli. Treatments were done in triplicate, and the percentage of feeding larvae was determined as described (Hawdon and Schad, 1990). To monitor feeding, 100 *μ*L of 2.5 mg mL^−1^ fluorescein-labelled bovine serum albumin (Sigma) was added to the wells at 24 h and incubated for 2–3 h at 37°C, 5% CO_2_. After incubation, the labelled larvae were washed with phosphate-buffered saline and examined by fluorescence microscopy. Feeding larvae, as evidenced by the presence of fluorescence in the lumen of the oesophagus and intestine, were counted and expressed as a percentage of the total larvae counted. A minimum of 50 total larvae were counted. To perform the larval activation inhibition assay (LAIA) to detect PYR resistance, WMD and BCR iL3 were incubated as described above together with increasing concentrations of PYR, and feeding determined at 24 h.

### Statistical analyses

Tests for statistical significance were conducted in GraphPad Prism (ver. 9, GraphPad Software, La Jolla CA USA). A 1-tailed *t*-test was used to calculate the significance between average BCR and WMD feeding behaviours in the LAIA. A pairwise ANOVA was used to compare feeding behaviours of BCR, KGR and WMD in the presence of serum stimulus or 8-bromo-cGMP. A 1-way ANOVA was used to compare the feeding behaviours of BCR and WMD in the presence of serum stimulus and various concentrations of pyrantel.

## Results

### Resistance assays

A member of the Microbiology, Immunology, and Tropical Medicine department at The George Washington University had recently adopted a 3.6-year-old retired racing greyhound (named ‘Bean Counter’) with a history of a hookworm infection that was refractory to treatment with several anthelmintic regimens. Our recent report (Kitchen *et al*., 2019), as well as that of others (Jimenez Castro *et al*., 2019, 2021), on multidrug-resistant hookworms isolated from greyhounds led us to predict the Bean Counter's hookworms were also multidrug resistant. To further investigate and characterize the isolate, a naïve beagle pup was infected with iL3 reared from Bean Counter's feces to establish the ‘BCR’ (Bean Counter-resistant) isolate in the lab.

**Figure 1.**
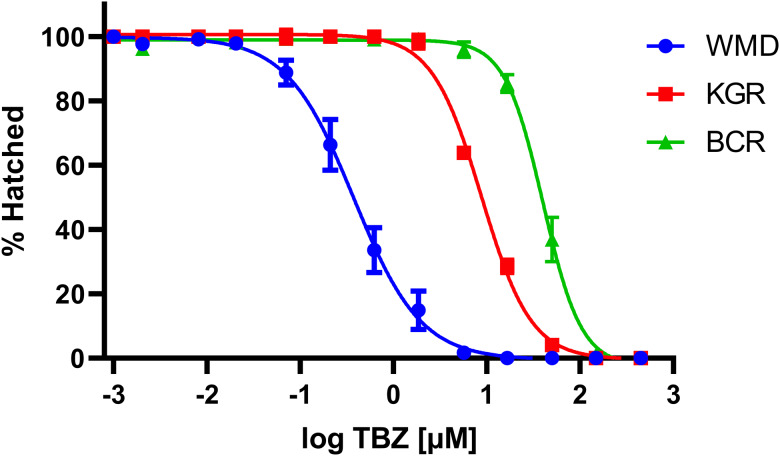
Dose–response curves of *Ancylostoma caninum* isolates to thiabendazole (TBZ) using the egg hatch assay (EHA). Each point represents a minimum of 6 replicates from 3 independent experiments (WMD *n* = 59, KGR *n* = 60, BCR *n* = 62). The curves were analysed using the sigmoidal dose–response (variable slope) function in GraphPad Prism (ver. 9) to generate the IC_50_ values reported in Table 1.

Resistance of the BCR isolate to the BZ anthelmintic TBZ was measured using an EHA. A distinct rightward shift along the *x*-axis in the dose–response curve is seen in both resistant isolates (KGR and BCR) when compared to the wild-type WMD (Fig. 1). The BCR isolate was over 4 times more resistant to TBZ than KGR, with an IC_50_ of 40.08 *μ*m compared to 8.76 *μ*m for KGR. The RR, calculated by dividing the IC_50_ of the resistant isolate by the IC_50_ of the susceptible (WMD) isolate, of BCR was 105.5 compared to 23.1 in KGR (Table 1, Fig. 1).

**Table 1.** Half maximal inhibitory concentration (IC_50_) and resistance ratios (RR) of *Ancylostoma caninum* WMD, KGR and BCR isolates to thiabendazole, ivermectin and pyrantel pamoate

	Thiabendazole			Ivermectin			Pyrantel			Moxidectin		
	IC50	RR	SE(*n*)	IC50	RR	SE (*n*)	IC50	RR	SE (*n*)	IC50	RR	SE (*n*)
WMD	0.38	–	0.0135 (59)	15.65	–	0.0171 (48)	13.73	–	0.0190 (48)	4.21	–	0.0285 (50)
KGR	8.76	23.1	0.0125 (60)	233.7	14.9	0.1115 (49)	18.39	1.3	0.0528 (50)	nd	nd	nd
BCR	40.08	105.5	0.0097 (62)	127.0	8.1	0.0379 (50)	21.09	1.5	0.0090 (49)	425.1	101	0.2432 (63)

TBZ, thiabendazole; IVM, ivermectin; PYR, pyrantel pamoate; WMD, susceptible isolate; KGR, previously reported resistant isolate; BCR, resistant isolate; SE, standard error of the logIC_50_ reported by Graphpad Prism (ver 9); *n* = average number of larvae per replicate. IC_50_ values (*μ*M) were generated by analysis of dose–response curves determined by larval development assays (LDA) and resistance ratio (RR) generated by dividing resistant isolate IC_50_ by WMD wild type IC_50_.

**Figure 2.**
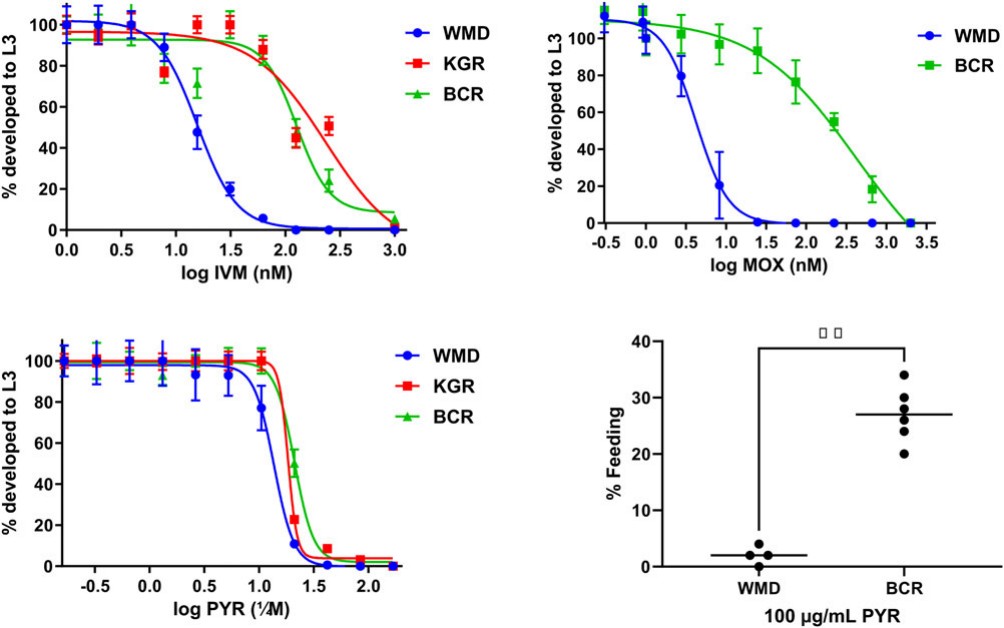
Dose–response curves of *Ancylostoma caninum* isolates WMD (sensitive), KGR (double resistant) and BCR (triple resistant) using a larval development assay (LDA) and a larval activation inhibition assay (LAIA). Dose–response curves to (A) ivermectin (IVM), (B) moxidectin (MOX) and (C) pyrantel (PYR) determined by LDA. The curves were analysed using the sigmoidal dose–response (variable slope) function in GraphPad Prism (ver. 9) to generate the IC_50_ values reported in Table 1. Each point represents a minimum of 6 replicates from 3 independent experiments. (D) *In vitro* larval activation inhibition assay (LAIA) for PYR resistance. Third-stage infective larvae (iL3) of susceptible WMD and resistant BCR isolates were incubated with 100 *μ*g mL^−1^ PYR overnight at 37°C, then assayed for activation. The mean of 4 (WMD) or 6 (BCR) replicates from 2 or 3 independent experiments were analysed by 1-tailed *t*-test in GraphPad Prism. **P < 0.01.

**Figure 3.**
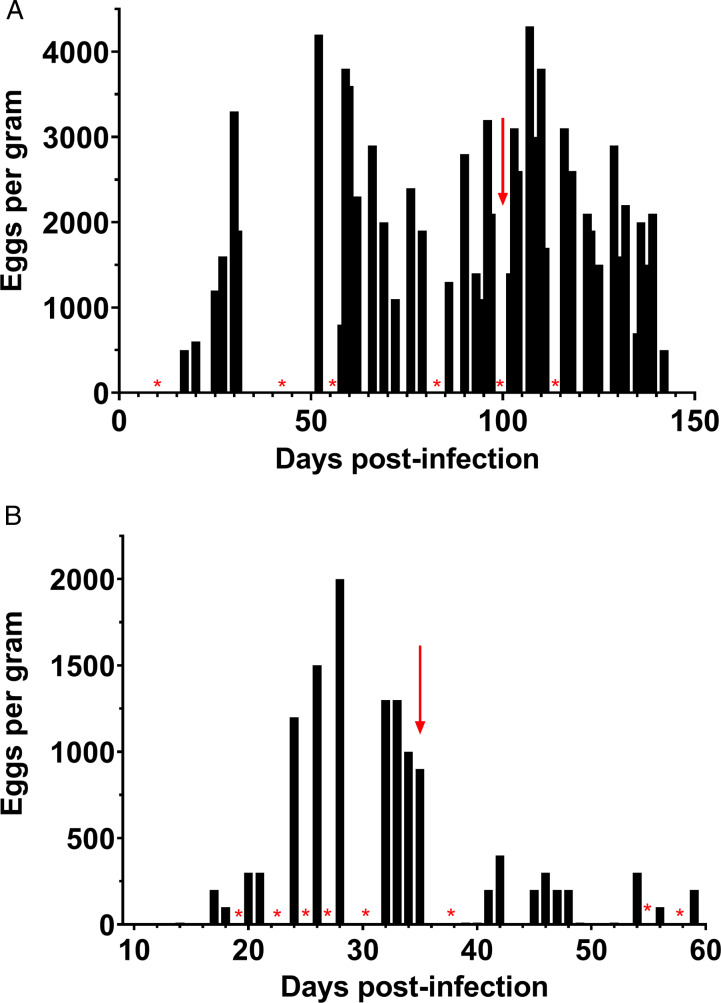
Fecal egg counts (FEC) in BCR and KGR isolates. (A) FEC were performed over the course of infection for a dog infected with the BCR hookworm isolate and (B) the KGR isolate to test for pyrantel resistance. The red arrow marks when pyrantel treatment was administered. BCR and KGR were treated at 101- and 37-days post-infection, respectively. Blank areas marked with red asterisks indicate days when the FEC was not measured.

Next, an LDA assay was used to determine if BCR was resistant to IVM, MOX and PYR. As shown in Fig. 2A, the dose–response curves for KGR and BCR are again shifted rightward, indicating that both isolates are resistant to IVM. The IC_50_ of the KGR isolate was 233.7 nm and the RR was 14.9, nearly twice that of the BCR with an IC_50_ of 127.0 nm and RR of 8.1 (Table 1). BCR worms were profoundly resistant to the MOX (IC_50_ 425.1 nm, RR 101), a newer member of the ML drug family (Fig. 2B). However, when BCR was tested for PYR resistance using the LDA, no significant resistance could be demonstrated (BCR RR = 1.5, KGR RR = 1.3) (Fig. 2C). The inability to detect PYR resistance using the LDA has been reported previously (Kopp *et al*., 2008; Jimenez Castro *et al*., 2019). Given the co-occurrence of PYR with BZ and IVM resistance in greyhounds, a FECRT was performed following PYR treatment. Treatment of a BCR-infected dog with a standard dose of PYR (5 mg kg^−1^) had no effect on the infection. The average FEC the week prior to treatment was 1950 EPG, while the average FEC on days 9–11 post-treatment was 2833 EPG (FECR = −45.2%). Conversely, treatment of a KGR-infected dog reduced the egg count significantly, from 1200 EPG pre-treatment to 233 EPG 10–12 days post-treatment (FECR = 80.6%) (Fig. 3). To confirm PYR resistance in BCR, an LAIA was used, which is a modification of the *in vitro* larval feeding inhibition assay (LFIA) (Kopp *et al*., 2008). As shown in Supplementary Fig. 1, concentrations of pyrantel greater than 20 *μ*g mL^−1^ significantly inhibited feeding of WMD L3 compared to BCR. The 100 *μ*g mL^−1^ concentration was selected for the assay because of the highly significant (*P* < 0.01) feeding difference between the resistant and susceptible isolates. At a PYR concentration of 100 *μ*g mL^−1^, 27 ± 5% of BCR L3 were feeding, whereas 2 ± 1.6% of WMD L3 fed (Fig. 2D). Together these results indicated that the BCR isolate is resistant to PYR in addition to TBZ, IVM and MOX.

### Sequencing of the BCR *β*-tubulin isotype 1 gene

**Figure 4.**
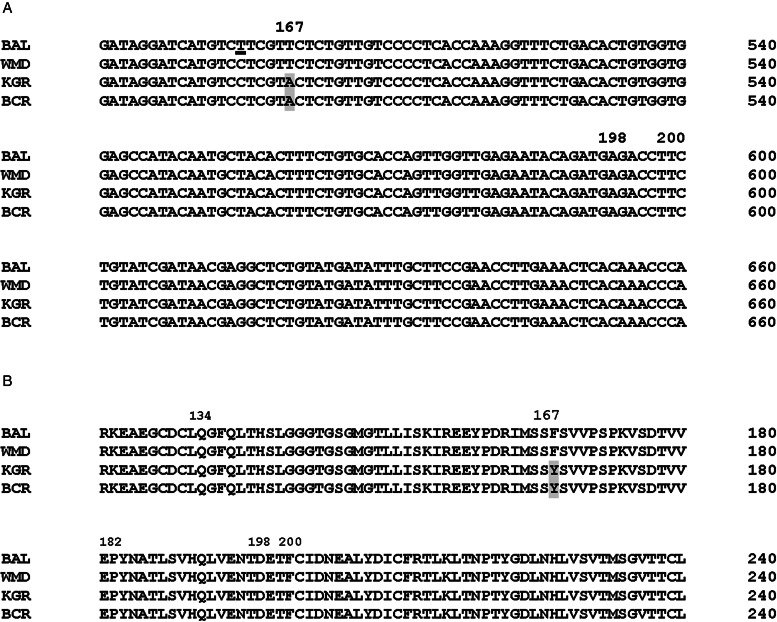
Sequences of *β*-tubulin isotype 1 cDNAs from 4 *Ancylostoma caninum* isolates. (A) DNA sequences. The non-synonymous mutation in codon 167 associated with benzimidazole resistance is shaded grey. A synonymous mutation in codon 165 of the BAL sequence is underlined. There were no mutations in codons 134, 182, 198 and 200, which have been associated with resistance to benzimidazoles, detected using this technique. (B) Protein translation of the relevant region of the *β*-tubulin isotype 1 sequence. The F167Y mutation is highlighted. BAL, Baltimore isolate reference genome (DQ059758; Bioproject PRJNA72585); WMD, thiabendazole-susceptible isolate (MH253570); KGR, thiabendazole-resistant isolate (MH253569); BCR, thiabendazole-resistant isolate (MZ889668).

BZ resistance in parasitic nematodes is most frequently associated with single nucleotide polymorphisms (SNPs) in 1 of 3 codons in the *β-*tubulin isotype 1 (F200Y, E198L, F167Y) (Kwa *et al*., 1993a, 1993*b*, 1994; Beech *et al*., 1994; Elard and Humbert, 1999; Silvestre and Cabaret, 2002; Ghisi *et al*., 2007; Kotze *et al*., 2012; Hahnel *et al*., 2018). More recent work has identified an SNP in another codon (Q134H) in the *β-*tubulin isotype 1 of *A. caninum* (Venkatesan *et al*., 2023). To date, all hookworm isolates resistant to benzimidazole anthelmintics have exhibited the F167Y or Q134H mutation (Jimenez Castro *et al*., 2019, 2021; Kitchen *et al*., 2019; Venkatesan *et al*., 2023). To determine if any of these mutations were present in BCR-resistant worms, the *tbb-iso-1* cDNA was amplified and sequenced and compared to the *A. caninum* Baltimore isolate (GenBank accession number DQ059758), our previous resistant isolate KGR (MH253569) (Kitchen *et al*., 2019) and our susceptible isolate WMD (MH253570) sequences. Thirteen synonymous mutations and a single non-synonymous SNP that changed TTC (Phe) to TAC (Tyr) in amino 167 of the BCR isolate were found (Fig. 4). This SNP has been associated with BZ resistance in several strongylid nematodes, including hookworms (Silvestre and Cabaret, 2002; Albonico *et al*., 2004; Schwenkenbecher *et al*., 2007; Vercruysse *et al*., 2011; Jimenez Castro *et al*., 2019, 2021; Kitchen *et al*., 2019), and imparted TBZ resistance to *C. elegans* edited to contain the corresponding mutation in the *ben-1* gene (Kitchen *et al*., 2019). No mutations were found at amino acid 134, 198 or 200 of *tbb-iso-1* in BCR using this sequencing approach.

Considering the relatively high frequency of the Q134H allele reported in canine populations, the limitations in our sequencing approach and large differences in RRs between KGR and BCR isolates (above), the recently sequenced genomes of WMD, KGR, BCR and BCR (P1) were interrogated for the presence of the Q134H allele. Examination of the *tbb-iso-1* gene for mutations revealed both the F167Y (TTC>TAC) and Q134H (CAA>CAT) alleles. The alleles were not observed together on the same haplotype, suggesting they occur in a *trans* configuration (Supplementary Fig. 2).

### Resistant allele frequencies

Previously, the frequency of the 167 resistant alleles (TAC) was found to be fixed at 100% in our KGR isolate (Kitchen *et al*., 2019). To determine the frequency of the TAC allele in BCR, a sensitive quantitative PCR assay was used to measure the relative ratio of the susceptible allele to the resistant allele (Germer *et al*., 2000; Schwenkenbecher *et al*., 2007; Kotze *et al*., 2012; Kitchen *et al*., 2019). The allele frequencies in WMD, KGR and the first passage of BCR were determined. As shown in Table 2, the frequency of the resistant allele in WMD was <1%, but 99.97% in KGR. In larvae recovered from the first BCR infection, the frequency of the resistant allele was 76.3%, indicating the presence of heterozygotes in the population. Next, the allele frequencies in the original Bean Counter iL3 from the same batch used to establish the BCR isolate were measured. The allele frequencies in these Bean Counter larvae were 44.1 and 55.9% for the susceptible and resistant allele, respectively. This indicates that the frequency of the TAC-resistant allele increased from 56 to 76% with passage of the isolate *in vivo* in the absence of anthelmintic treatment.

**Table 2.** Frequency of wild-type and resistant *β*-tubulin F167Y allele in hookworm isolates

	gDNA pool	qPCR target	Ct Mean	Ct s.d.	ΔCt	Sensitive allele frequency (%)	Resistant allele frequency (%)
Expt.1	WMD	Sensitive	24.02	0.067	−14.92	99.996	0.003
		Resistant	38.94	0.000			
	KGR	Sensitive	35.15	0.359	11.50	0.034	99.97
		Resistant	23.65	0.057			
	BCR	Sensitive	26.25	0.318	1.69	23.66	76.34
		Resistant	24.56	0.219			
Expt. 2	WMD	Sensitive	24.94	0.210	−29.58	99.99	1.25 × 10^−7^
		Resistant	54.56	6.689			
	KGR	Sensitive	31.36	0.059	6.40	1.17	98.83
		Resistant	24.96	0.296			
	Bean	Sensitive	28.11	0.072	0.34	44.12	55.88
	Counter	Resistant	27.77	0.554			

Allele frequencies were calculated using the formula frequency = 1/(2^ΔCt^ + 1). Cycle threshold (Ct) values are the mean of 4 technical replicates. ΔCt = Ct_−sen_–Ct_−res_. WMD, susceptible isolate; KGR, double-resistant isolate; BCR, triple resistant isolate; Bean Counter, larvae from the infected greyhound that was the source of isolate BCR.

Based on the number of reads that support each allele (i.e. allele counts) in our genomic sequence data, the allele frequency of tubulin mutations in each of the isolates was estimated. The frequency of the TAC resistance mutation in codon 167 was 96.4% (54/56 reads) in KGR and 84.0% (21/25) in BCR. As the number of reads in the BCR sequences was reduced due to bacterial contamination, we also estimated the TAC allele frequency in BCR (P1), which was derived from iL3 collect from the first passage of BCR following treatment with PYR for the FECRT. The frequency of the TAC allele was 81.8% (99/121) in BCR (P1). The allele frequencies from the genomic sequences were similar to the allele frequencies determined by quantitative PCR (99.97 and 76.3%, respectively). The frequency of the TAC mutation in WMD was zero (0/55). The CAT resistance mutation in codon 134 was present in 16.0% (4/25) of the reads in BCR and 18.2% (26/143) of the reads in BCR (P1) but was absent (0/53 and 0/54) in KGR and WMD, respectively.

### Altered activation phenotype

**Figure 5.**
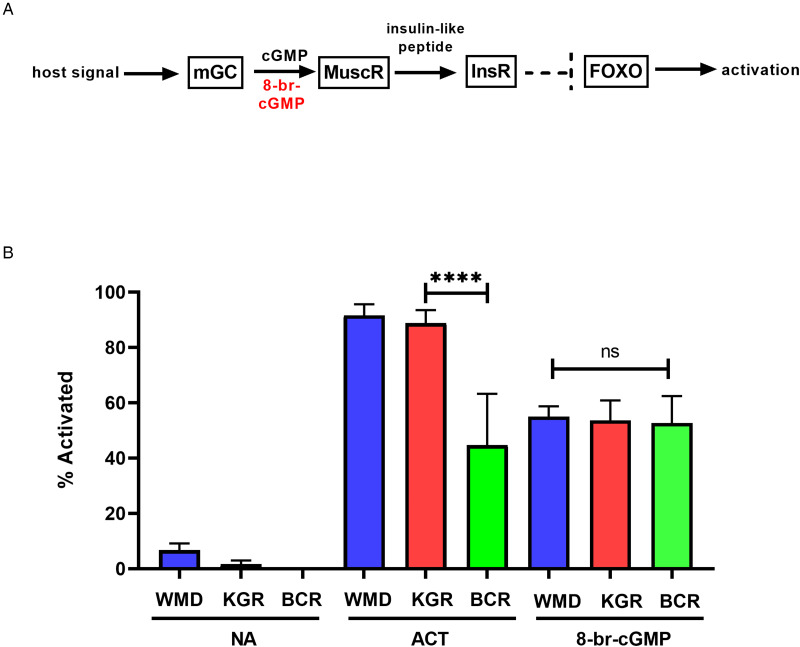
Altered feeding response in pyrantel-resistant BCR isolate. (A) General activation pathway. (B) Hookworm iL3 from each isolate were incubated in medium alone (NA), serum stimulus (ACT) or 15 mm 8-bromo-cGMP for 24 h, after which the number of activated (feeding) iL3 was determined. Experiments were done in triplicate and repeated twice, so that each value is the mean ± s.d. of 9 replicates, and analysed by pairwise ANOVA. ****P < 0.0001. mGC, membrane guanylyl cyclase; MuscR, muscarinic receptor; InsR, insulin receptor, FOXO, forkhead transcription factor.

Feeding by infective L3 of hookworms and other nematodes in response to host-like stimuli *in vitro* has been termed larval activation (Hawdon and Schad, 1990, 1991*a*). Due to its similarity to recovery from the dauer stage in *C. elegans*, hookworm larval activation is believed to represent an early step in the transition to parasitism that occurs during infection of a permissive host (Hawdon and Schad, 1991a; Hawdon and Hotez, 1996). The activation pathway in hookworms is highly conserved with recovery from the dauer stage in *C. elegans*, and is mediated by both cGMP and ILS pathways (Tissenbaum *et al*., 2000; Hawdon and Datu, 2003; Brand and Hawdon, 2004; Gao *et al*., 2009; Kiss *et al*., 2009; Gelmedin *et al*., 2011) (Fig. 5A). When BCR iL3 were tested in our activation assay, their maximum feeding level was significantly lower (approximately 40–50%) than the feeding level of the wild-type WMD and the double-resistant KGR isolates (Fig. 5B). Exogenous cGMP (as 8-bromo-cGMP) initiated feeding similarly in all isolates (Fig. 5B), indicating that the signalling pathway downstream of the cGMP step is functioning normally in BCR worms, and that the defect causing decreased activation is upstream, likely in the membrane guanylyl cyclase (mGC) itself, as mGC is believed to be the first molecule in the activation pathway (Fig. 5A) (Murakami *et al*., 2001).

## Discussion

Domestic dogs are the most common companion animal, with an estimated 700 million worldwide (Hughes and Macdonald, 2013). This popularity comes with the potential for zoonotic exposure of people to canine parasites and risk of disease from these parasites. Hookworms are one of the greatest zoonotic concerns presenting significant risk to human health (Otranto *et al*., 2017). The most common zoonotic species, *A. caninum*, is found in wild and domestic canids around the world. *Ancylostoma caninum* causes follicular cutaneous *larva migrans* (CLM), an intensely pruritic skin infection, in humans exposed to infective larvae. While the prevalence of CLM in the United States is unknown, it is thought to be second behind pinworm among helminth infections in developed countries (Robles and Habashy, 2020). Other zoonotic diseases caused by hookworms include eosinophilic enteritis resulting from a single immature adult worm establishing in the intestine, and diffuse unilateral subacute neuroretinitis, a serious infection caused by infective larvae migrating through the eye (Hawdon and Wise, 2021). Patent infections in humans infected with *A. caninum* have been recently identified, suggesting that infections in humans are more common than previously thought (Ngcamphalala *et al*., 2019; Furtado *et al*., 2020; Hawdon and Wise, 2021).

Pet dog ownership in the United States is at the highest level ever reported; 45% of households own a dog, totalling 83 to 89 million animals (American Veterinary Medical Association, 2022). *Ancylostoma caninum* is the most prevalent and significant intestinal nematode of dogs in the United States (Little *et al*., 2009), and its prevalence has increased significantly over the last 5 years (Drake and Carey, 2019; Sweet *et al*., 2021). Heavy infection causes severe, often fatal anaemia in puppies, and malnutrition and failure to thrive in older dogs. A recent retrospective survey found that hookworms were the most prevalent canine helminth parasite in 9 US states at approximately 5.6%, ranging as high as 11.9% in Georgia (Sobotyk *et al*., 2021). Hookworm prevalence has been increasing (Drake and Carey, 2019; Nagamori *et al*., 2020), possibly reflecting the emergence of multiple anthelmintic-resistant hookworm isolates like BCR reported here in greyhounds and spreading to the general pet dog population (Jimenez Castro *et al*., 2019, 2021; Kitchen *et al*., 2019; Jimenez Castro and Kaplan, 2020; Balk *et al*., 2023; Leutenegger *et al*., 2023; Venkatesan *et al*., 2023). Increasing prevalence of MADR *A. caninum* has serious implications both for canine companion health and for zoonotic infections (Hawdon and Wise, 2021). Indeed, the BCR isolate is profoundly resistance to the 3 classes of anthelmintic that are currently used for treatment of canine and zoonotic hookworm infections, which will complicate treatment of hookworm infections should they become widespread.

The IC_50_ of BCR to TBZ is nearly 5 times that of our previously reported resistant KGR isolate in an EHA (40.1 *vs* 8.76 *μ*m, RR 106 *vs* 23). The BCR is significantly more resistant than most of the previously published MADR *A. caninum* isolates that had not undergone selection in the lab (Jimenez Castro *et al*., 2019, 2021). Increased resistance of BCR relative to KGR is interesting, as frequency of the resistant F167Y allele is lower in the BCR isolate (76.3%) than in KGR, where it is essentially fixed (99.97%). A possible explanation for this difference is the presence of a recently identified mutation in codon 134 (CAA>CAT, Q134H) found at low frequency in the BCR, but not the KGR, isolate. However, introduction of this mutation into *C. elegans ben-1* conferred a similar level of resistance as the *ben-1* null allele (Venkatesan *et al*., 2023). In *C. elegans*, additional mutations in canonical residues of the *ben-1* gene have been identified that confer resistance (Dilks *et al*., 2020, 2021), and there are other non-tubulin mutations suspected to influence BZ resistance (Zamanian *et al*., 2018), although mutagenesis screens in *C. elegans* yielded either no mutations outside of the *ben-1* gene, or a single non-tubulin mutation that was only weakly resistant (Driscoll *et al*., 1989; Pallotto *et al*., 2022).

Unlike TBZ resistance, resistance of the BCR isolate to IVM in the LDA was 54% lower than KGR (127.0–233.7 *μ*m), with IC_50_ RRs of 8.1 and 14.9, respectively. Previously, a higher IVM RR for KGR was reported (Kitchen *et al*., 2019), which was likely due to technical differences in performance of the assays. The IC_50_ and RR values for IVM reported herein are more similar to those reported by Jimenez Castro *et al*. (2019). The BCR isolate is highly resistant to MOX, a newer, more potent macrocyclic lactone drug. Resistance to MOX appears to develop in a stepwise process, beginning with resistance to IVM (Kaplan *et al*., 2007). For example, IVM-resistant, MOX-naïve *Haemonchus contortus* were found to be susceptible to MOX (Craig *et al*., 1992; Oosthuizen and Erasmus, 1993). However, IVM-resistant populations quickly obtain resistance to MOX with regular treatment. In addition to BCR, multiple MADR *A. caninum* isolates from greyhounds exhibit high levels of MOX resistance (Jimenez Castro *et al*., 2021), most likely as a result of treatment of adopted greyhounds with MOX-containing heartworm preventative formulations. We obtained infective larvae from the greyhound Bean Counter and established the BCR isolate prior to any treatments with MOX-containing formulations by his adopter. We cannot rule out exposure to MOX prior to his adoption, as medical records for those periods were unavailable. In any case, resistance to MOX further complicates treatment of dogs or humans infected with MADR hookworms.

Previous isolates have been reported to be resistant to PYR in addition to BZ and MLs (Jimenez Castro *et al*., 2019, 2021, 2022). Our LDA assay using PYR failed to discriminate between the susceptible and BCR isolates (Fig. 2C). Lack of discrimination in LDA for PYR has been reported previously, necessitating the use of the FECRT to confirm resistance to PYR (Kopp *et al*., 2007; Jimenez Castro *et al*., 2019; Jimenez Castro and Kaplan, 2020). The FECRT compares pre- and post-treatment mean egg counts to determine anthelmintic susceptibility. Our results indicated that the BCR isolate was highly resistant to PYR, as illustrated by a negative FECR, whereas the KGR isolate was much more susceptible, with an FECR of 80.6%. There are several disadvantages to the FECR test, most notably the need to infect dogs. Furthermore, the low sample size (*n* = 1) and other deviations from recommended guidelines for FECR tests (Kaplan *et al*., 2023) used here limit interpretation somewhat. In addition to the LDA, other *in vitro* tests have been used in attempts to determine PYR sensitivity, with mixed results (Kopp *et al*., 2008). To confirm our results, a larval arrested morphology assay was attempted first, which in our hands was difficult to interpret (not shown). Next, an LAIA, which is a modified version of the LFIA described by Kopp *et al*. (2008), was used. The LFIA, originally adapted from the hookworm activation assay (Hawdon and Schad, 1990), uses the inhibition of iL3 feeding, or activation, as an indicator of PYR sensitivity. In the modified LAIA, the iL3 and increasing PYR concentrations were incubated together initially and feeding determined approximately 24 h later rather than adding PYR following the initial 24 h incubation as described (Kopp *et al*., 2008). Using this assay, a significant difference (*P* < 0.01) between feeding by BCR and WMD larvae was found at 100 *μ*g mL^−1^ PYR, confirming that BCR is resistant to PYR.

When developmentally arrested hookworm iL3 enter a permissive host, a host-specific signal initiates the resumption of development, which culminates in the adult stage in the intestine. Early steps in this process can be modelled *in vitro*. When *A. caninum* iL3 are exposed to a low molecular weight canine serum fraction and a glutathione analogue (F + G), they resume feeding, or ‘activate’, within 6 h, and reach 90% of the iL3 population feeding by 16 h (Hawdon and Schad, 1992; Hawdon and Hotez, 1996). Activation is thought to represent an early step in infection and is biologically analogous to recovery from the developmentally arrested dauer stage of the model nematode *C. elegans* (Hawdon and Schad, 1991a; Hawdon and Hotez, 1996). Dauer arrest in *C. elegans* is facultative, occurring in response to overcrowding, mediated by the concentration of ascaroside pheromones, and lack of food (Hu, 2007; Ludewig and Schroeder, 2013). Dauer recovery occurs in response to food and lower nematode density and is regulated by the mGC DAF-11 expressed in amphidial neurons ASI, ASJ and ASK (Hanna-Rose and Han, 2000). DAF-11 binds a bacterial food signal, initiating cGMP synthesis and subsequent signalling through a conserved ILS pathway to cause recovery (Tissenbaum *et al*., 2000; Murakami *et al*., 2001; Chung *et al*., 2013). The dauer pathway is well-studied genetically and biochemically, and the molecular components of the pathway have been identified (Riddle and Albert, 1997; Hu, 2007). Furthermore, the pathway is conserved in GINs, and hookworm iL3 activation is mediated by ILS (Tissenbaum *et al*., 2000; Brand and Hawdon, 2004; Gao *et al*., 2009; Kiss *et al*., 2009; Wang *et al*., 2009; Gelmedin *et al*., 2011; Zhi *et al*., 2012) and cGMP signalling (Hawdon and Datu, 2003) as in *C. elegans* (Birnby *et al*., 2000).

When the BCR isolate iL3 were tested for their ability to activate *in vitro*, they resumed feeding in response to the activation stimulus at levels approximately half that of the WMD and KGR isolates. This altered phenotype is likely caused by a defect at or upstream of the DAF-11/mGC step, as BCR activation in response to exogenous cGMP is unaffected (Fig. 5). While the activation signal is thought to bind to the extracellular domain of the hookworm mGC, it is possible that the signal binds to an upstream G protein-coupled receptor which subsequently signals through the mGC.

Evolutionary theory assumes that the evolution of drug resistance is associated with a fitness trade-off, usually one that is deleterious in the absence of selection (Durão *et al*., 2018; Hodgkinson *et al*., 2019). The altered activation phenotype may be evidence of such a trade-off whereby a defect or change in sensitivity in the mGC is a consequence of the mutations that also impart resistance. This defect could render the resistant population of hookworms less capable of resuming feeding in the presence of host serum compared to the susceptible strain. If activation is required for the transition to parasitism, then the depressed activation phenotype described here may affect the ability of MADR hookworms to infect their host. While these results are from a single resistant isolate, if this altered phenotype is found to be consistently associated with, or the result of, multi-anthelmintic resistance, it could serve as a marker for resistance, particularly if it is associated with resistance to a particular anthelmintic. If so, it may provide useful insights into anthelmintic resistance in *A. caninum*. In any case, further research into the biology and mechanism of anthelmintic resistance in hookworms is warranted.

## Supporting information

McKean et al. supplementary material 1 McKean et al. supplementary material

McKean et al. supplementary material 2 McKean et al. supplementary material

## Data Availability

Pool-seq data have been deposited into the Sequence Read Archive database (SRA, https://www.ncbi.nlm.nih.gov/sra) under BioProject PRJNA72585. The accession numbers are SRR26411280 (WMD), SRR26411281 (KGR), SRR26411283 (BCR) and SRR26411282 (BCR P1).

## Supplementary material

The supplementary material for this article can be found at https://doi.org/10.1017/S0031182023001385.
