# Supplementary figures and images for: Altered larval activation response associated with multidrug resistance in the canine hookworm Ancylostoma caninum
Source: Parasitology. 2024 Jan 2;151(3):271–81. doi: 10.1017/S0031182023001385 (PMC11007283; doi:10.1017/S0031182023001385)

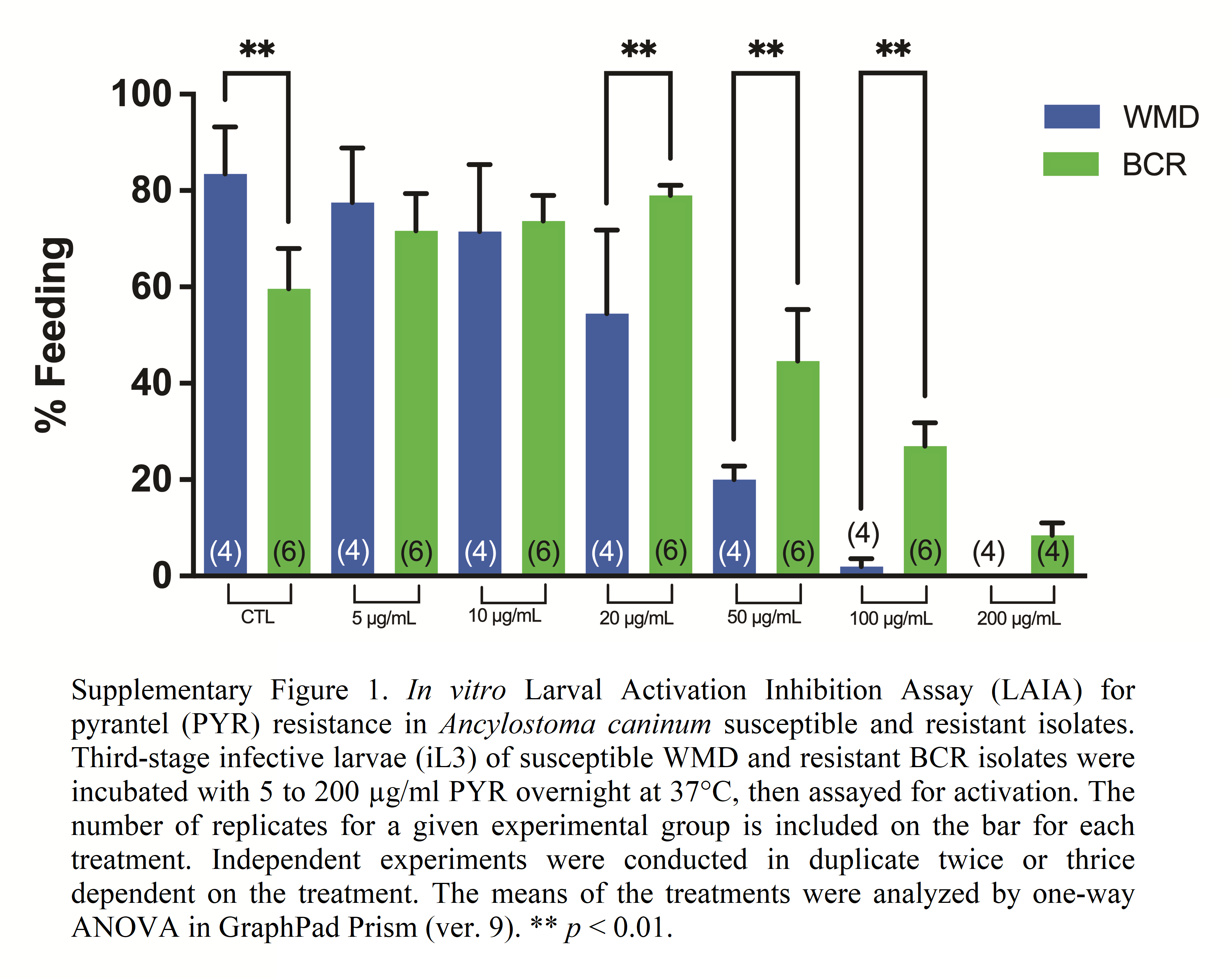

Supplement: McKean et al. supplementary material 1 — McKean et al. supplementary material [file S0031182023001385sup001.tif]

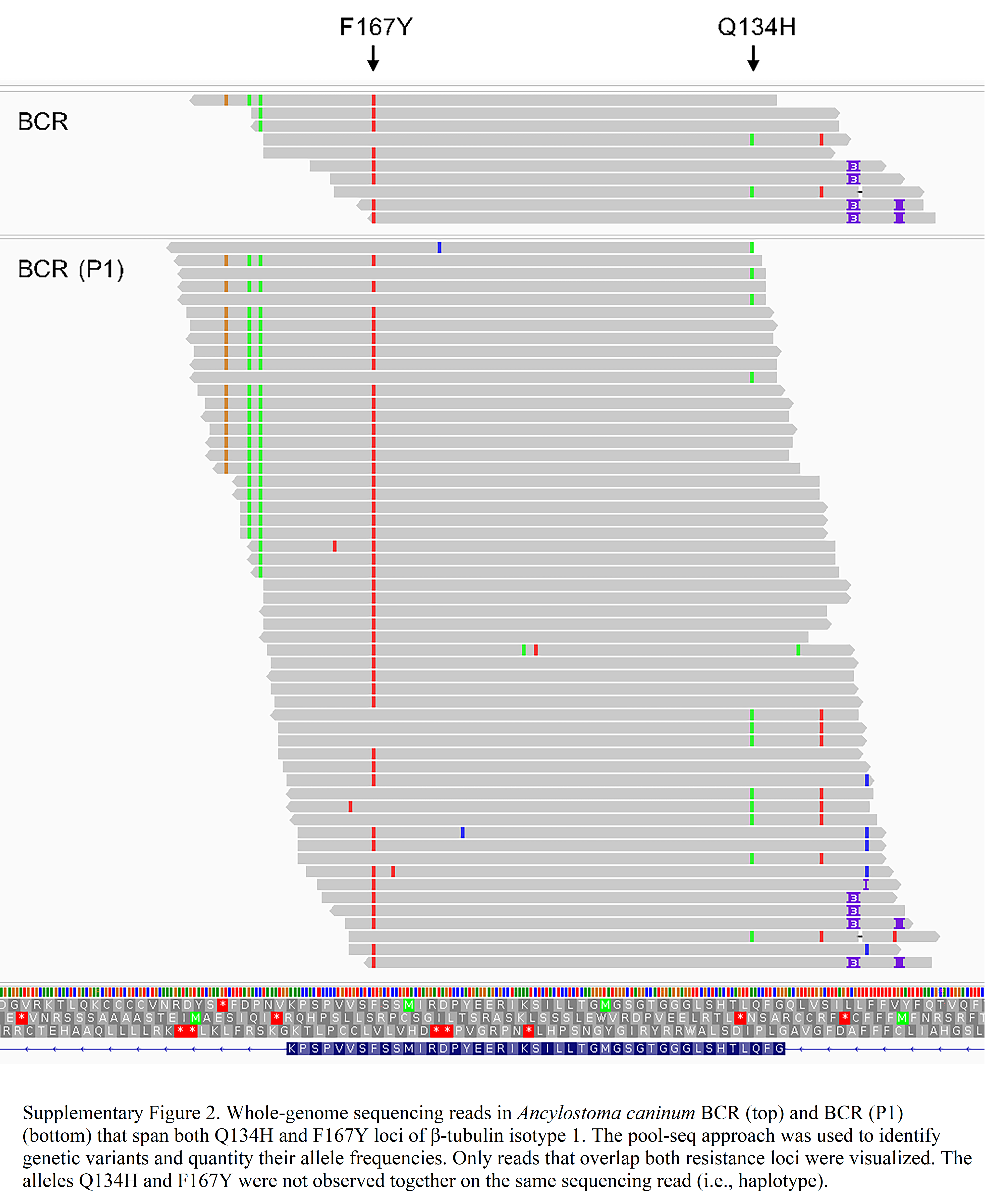

Supplement: McKean et al. supplementary material 2 — McKean et al. supplementary material [file S0031182023001385sup002.tif]
